# A Multimodal Fusion Analysis of Pretreatment Anatomical and Functional Cortical Abnormalities in Responsive and Non-responsive Schizophrenia

**DOI:** 10.3389/fpsyt.2021.737179

**Published:** 2021-12-01

**Authors:** Chenyang Yao, Na Hu, Hengyi Cao, Biqiu Tang, Wenjing Zhang, Yuan Xiao, Youjin Zhao, Qiyong Gong, Su Lui

**Affiliations:** ^1^Department of Radiology, Huaxi Magnetic Resonance Research Center, West China Hospital, Sichuan University, Chengdu, China; ^2^Department of Radiology, West China Hospital, Sichuan University, Chengdu, China; ^3^Department of Imaging Medicine, Inner Mongolia Autonomous Region People's Hospital, Hohhot, China; ^4^Center for Psychiatric Neuroscience, Feinstein Institute for Medical Research, Manhasset, NY, United States; ^5^Division of Psychiatry Research, Zucker Hillside Hospital, Glen Oaks, NY, United States

**Keywords:** mCCA + jICA, multimodal fusion, functional MRI, structural MRI, schizophrenia, antipsychotic medication, treatment-resistant schizophrenia

## Abstract

**Background:** Antipsychotic medications provide limited long-term benefit to ~30% of schizophrenia patients. Multimodal magnetic resonance imaging (MRI) data have been used to investigate brain features between responders and nonresponders to antipsychotic treatment; however, these analytical techniques are unable to weigh the interrelationships between modalities. Here, we used multiset canonical correlation and joint independent component analysis (mCCA + jICA) to fuse MRI data to examine the shared and specific multimodal features between the patients and healthy controls (HCs) and between the responders and non-responders.

**Method:** Resting-state functional and structural MRI data were collected from 55 patients with drug-naïve first-episode schizophrenia (FES) and demographically matched HCs. Based on the decrease in Positive and Negative Syndrome Scale scores from baseline to the 1-year follow-up, FES patients were divided into a responder group (RG) and a non-responder group (NRG). Gray matter volume (GMV), fractional amplitude of low-frequency fluctuation (fALFF), and regional homogeneity (ReHo) maps were used as features in mCCA + jICA.

**Results:** Between FES patients and HCs, there were three modality-specific discriminative independent components (ICs) showing the difference in mixing coefficients (GMV-IC7, GMV-IC8, and fALFF-IC5). The fusion analysis indicated one modality-shared IC (GMV-IC2 and ReHo-IC2) and three modality-specific ICs (GMV-IC1, GMV-IC3, and GMV-IC6) between the RG and NRG. The right postcentral gyrus showed a significant difference in GMV features between FES patients and HCs and modality-shared features (GMV and ReHo) between responders and nonresponders. The modality-shared component findings were highlighted by GMV, mainly in the bilateral temporal gyrus and the right cerebellum associated with ReHo in the right postcentral gyrus.

**Conclusions:** This study suggests that joint anatomical and functional features of the cortices may reflect an early pathophysiological mechanism that is related to a 1-year treatment response.

## Introduction

Antipsychotic medications provide limited long-term therapeutic benefits to only 30% of patients with schizophrenia (1, 2). In patients with first-episode schizophrenia (FES), acute response to antipsychotic treatment has been widely studied and typically shows favorable results (3). However, long-term outcomes seem to be more variable given the chronic course of the illness (4), strongly requiring further exploration for their predictors.

Neuroimaging has been used to investigate brain features between responders and non-responders to antipsychotic treatment and their longitudinal changes in schizophrenia patients (5–11). Magnetic resonance imaging (MRI) studies of short-term therapeutic outcomes have found differences in gray matter volume (GMV), cortical thickness, and resting-state brain function between responders and non-responders. Patients with a poorer response to antipsychotic treatment often exhibit lower GMV and/or thinner cortices in the frontal (8, 12, 13), temporal (3), postcentral (3), occipital (6), and calcarine cortices (14), and a smaller hippocampus (15). A recent study of antipsychotic-naïve patients showed that smaller baseline GMV in the insula and inferior frontal gyrus predicted limited improvements in positive and disorganization symptoms at the 1-year follow-up (16). In functional MRI studies on therapeutic effects in drug-naïve FES patients, positive correlations were found not only between the elevated fractional amplitude of low-frequency fluctuation (fALFF) in the putamen and improvements in positive symptoms after 8 weeks of treatment, but also between the fALFF reduction in the putamen after 1 week of treatment and improvements in positive symptoms after 8 weeks of treatment (17, 18). Another study found the regional homogeneity (ReHo) levels in the precuneus and superior medial prefrontal cortex could predict symptom improvement after 8 weeks of treatment with olanzapine plus psychotherapy (19). Together, evidence has suggested that several anatomical or functional MRI features may help illustrate treatment outcomes in schizophrenia patients. However, little is known about the combined performance of these multimodal measurements in studying longer-term (1-year clinical outcomes) responses to antipsychotic medications.

To fuse MRI data across modalities, a model of multiset canonical correlation and joint independent component analysis (mCCA + jICA) has been developed (20). Specifically, mCCA + jICA is a multimodal fusion analytical method to investigate specific and shared imaging features among multiple MRI modalities and to identify interrelated abnormalities across modalities. It allows the study of psychotic disorders at multiple analytic levels, which may help integrate information derived from different MR modalities (21). Previous studies have revealed differences in gray matter, white matter, and cerebrospinal fluid (21), and differences in GMV and dynamic functional connectivity in patients with schizophrenia (22, 23). Most of these studies were multimodal fusion studies from the perspective of the diagnosis of schizophrenia, and few studies have further explored which brain regions in patients are of significance in identifying patients' responses to treatment.

The present study aimed to investigate the differences in multimodal MRI features (GMV, fALFF, and ReHo) between patients with drug-naïve FES schizophrenia and healthy controls (HCs) and between treatment responders and non-responders. We hypothesized that patients with poorer clinical outcomes would differ from those with favorable outcomes in pretreatment joint anatomical and functional brain features that were identified with mCCA + jICA.

## Methods

### Study Participants

The study was approved by the local research ethics committee. Written informed consent was obtained from all participants. In all, 55 patients with drug-naïve FES were recruited from the Mental Health Center of our hospital, and 55 HCs were recruited from local communities. The sample size was determined by referring to a similar study using the same methods, in which 19 patients and 21 HCs were included (21). The diagnostic criteria of schizophrenia met the Structured Clinical Interview for DSM-IV (SCID), which was confirmed by the consensus of two psychiatrists. Patients had no Axis I psychiatric disorders other than schizophrenia, and HCs had no history of Axis I disorders or first-degree relatives with a history of psychiatric illness. The exclusion criteria for all participants were significant systemic disorders, neurological illness, substance abuse or dependence, pregnancy, or MRI contraindications. The patients and HCs were all right-handed, and the two groups were matched for age, sex, and education years.

At baseline, no patients had previously received antipsychotic treatment or other psychiatric medications. After MR scans and symptom assessments at baseline, all patients were treated with second-generation antipsychotic medications, and particular drug and dosage choices were determined by treating psychiatrists. During the 1-year follow-up, 85.5% (47/55) of patients had received a single antipsychotic drug, and the others had received multiple drugs. Meanwhile, 14.5% (8/55) received a single serotonin reuptake inhibitor. Antipsychotic and the additional drugs used are shown in Table 1. Daily dosages of antipsychotic drugs were converted into chlorpromazine (CPZ) equivalents (24). The severity of psychiatric symptoms was assessed with the Positive and Negative Syndrome Scale (PANSS) both at baseline and at the 1-year follow-up. The percentage reduction in PANSS at follow-up was calculated as follows:


$$\begin{array}{l} \frac{PANSS_{baseline}-PANSS_{follow-up}}{PANSS_{baseline}-30}\times 100\%. \end{array}$$

**Table 1:** Antipsychotic and additional drugs used in FES patients.

**Drugs**	**Total number of patients**	**RG**	**NRG**
Risperidone^a^	28	19	9
Quetiapine^d^	8	6	2
Sulpiride^b^	3	3	0
Aripiprazole^e^	3	2	1
Olanzapine^c^	2	2	0
Clozapine^a^ + sulpiride^b^	2	2	0
Quetiapine^d^	2	1	1
Paliperidone^a^	1	1	0
Clozapine^a^ + aripiprazole^e^	1	1	0
Clozapine^a^ + risperidone^a^	1	1	0
Sulpiride^b^ + olanzapine^c^	1	1	0
Olanzapine^c^ + quetiapine^d^	1	0	1
Risperidone^a^ + sulpiride^b^	1	1	0
Risperidone^a^ + clozapine^a^ + sulpiride^b^ + quetiapine^d^	1	1	0
Fluoxetine	5	3	2
Paroxetine	2	2	0
Clomipramine	1	1	0

*Drugs are categorized according to the Neuroscience-based Nomenclature (NbN) classification. RG, responder group; NRG, non-responder group*.

a *Dopamine, serotonin, and noradrenaline receptor antagonist (D2, 5-HT2, NE alpha-2)*.

b *Dopamine receptor antagonist (D2)*.

c *Dopamine and serotonin receptor antagonist (D2, 5-HT2)*.

d *Dopamine and serotonin receptor antagonist (D2, 5-HT2) and noradrenaline reuptake inhibitor (NET) (metabolite)*.

e *Dopamine and serotonin receptor partial agonist (D2, 5-HT1A)*.

A cutoff of at least 50% reduction was considered the criterion of a treatment response (25). Accordingly, the patients were divided into two groups, namely, the responder group (RG) (*n* = 40) and the non-responder group (NRG) (*n* = 15).

### Image Acquisition

The participants underwent brain scans at baseline using a 3T MRI system (EXCITE; General Electric, Milwaukee, Wisconsin) with an 8-channel phased-array head coil. High-spatial resolution T1-weighted images were acquired with a three-dimensional spoiled gradient-recalled sequence (repetition time, 8.5 ms; echo time, 3.4 ms; flip angle, 12°; field of view, 240 mm × 240 mm). An acquisition matrix comprising 256 readings of 128 phase-encoding steps yielded 156 contiguous coronal slices with 1-mm slice thickness. The final matrix was automatically interpolated in-plane to produce an in-plane resolution of 0.47 mm × 0.47 mm. Resting-state functional MRI was obtained with a gradient-echo echo-planar imaging sequence (repetition time, 2,000 ms; echo time, 30 ms; flip angle, 90°; slice thickness, 5 mm; matrix, 64 × 64; field of view, 240 mm × 240 mm; voxel size, 3.75 mm × 3.75 mm × 5 mm). Each brain volume comprised 30 axial slices, and each functional run contained 200 image volumes.

### Image Preprocessing and Feature Calculation

Image data were preprocessed with Data Processing Assistant for Resting-State fMRI (DPARSF) software (version 5.0; http://rfmri.org/DPARSF). This is a “pipeline” data analysis toolbox based on Statistical Parametric Mapping (SPM) and Resting-State fMRI Data Analysis Toolkit (REST). Compared with SPM, the procedures and methods applied to DPARSF are similar but easier to be operated in a pipeline manner. High-spatial resolution T1-weighted images were segmented into gray matter maps, which were then normalized to the Montreal Neurological Institute (MNI) space. To suppress noise and effects due to residual differences in anatomy during inter-subject averaging, Gaussian kernel smoothing was conducted with the full width at half maximum (FWHM) of 4 mm × 4 mm × 4 mm by using the Diffeomorphic Anatomical Registration Through Exponentiated Lie Algebra (DARTEL) algorithm (26). The resultant modulated images representing the volumes were used in the subsequent procedures, as they corrected for image distortions during spatial normalization (27). The main procedures of resting-state functional MRI included the removal of the first 10 time points, slice timing and head motion correction, realignment, segmentation, nuisance covariate regression, spatial normalization (voxel size, 3 mm × 3 mm × 3 mm), filtering (bandpass; calculated as the averaged square root of the power spectrum within a frequency range of 0.01–0.08 Hz), smoothing (FWHM, 4 mm × 4 mm × 4 mm), and linear trend removal. The fALFF and ReHo were extracted by the built-in DPARSF functions.

### MCCA + JICA Analysis

The mCCA + jICA algorithm implemented in the fusion ICA toolbox (FIT; version 2.0e; http://mialab.mrn.org/software/fit) was used to integrate imaging features from the three modalities. This algorithm runs in a synergistic scheme. Briefly, mCCA extracts mixing brain matrices to generate flexible correlations across modalities, assisting jICA in determining independent components (ICs) simply and precisely to the greatest extent. Here, the correlations between modalities are considered and weighted extensively, no matter whether they are strong or weak, or whether they share or differ completely. This ensures maximum independence between modalities. Complementarily, jICA improves source separation to decompose the mixing matrix into ICs. This fusion model is thus capable of examining full correspondence of the N-way brain datasets and optimizing for both flexibility in intermodal linkages and high capability of source separation (28). In the mCCA, GMV, fALFF, and ReHo images of each subject were transformed into a one-dimensional matrix to construct a feature matrix with the dimensions of the number of subjects by the number of voxels modeled as a product of the mixing profile and associated components (dimension reduction). Subsequently, in the jICA, the concatenated associated component matrix with the dimensions of the number of components by the sum of the number of voxels across imaging modalities was modeled by the demixing matrix and the joint independent components (29).

In our study, the mCCA + jICA analysis of multimodal features was conducted between the FES patients and HCs and then between the RG and NRG. First, the GMV, fALFF, and ReHo maps (feature maps) were reshaped for each participant and normalized with the same average sum-of-squares to ensure equal ranges across maps. Second, normalized maps were input into an mCCA + jICA model. Here, three-dimensional maps were reconstructed into a one-dimensional vector and superimposed to form a theme, which was composed of a voxel matrix (28). Third, the mean sum-of-squares was computed across all voxels and all patients for each modality, followed by normalization to reach modality-wise equality. Fourth, normalized features were reduced through mCCA and decomposed into ICs via jICA. The determined ICs were converted to Z-scores and masked with a brain template, the automated anatomical labeling (AAL) atlas. Only those with |Z| above 3.5 were displayed. These procedures were applied to both the responders vs. non-responders and the FES patients vs. HCs.

The mixing coefficient of an IC was extracted within each modality. A higher mixing coefficient indicated that the corresponding IC was expressed more in the experimental group than in the control group. The outliers were removed, defined as the values more than twice the interquartile range. Two-sample *t*-tests were performed on mixing coefficients to identify group differences in ICs. *P*-values below 0.05 were considered statistically significant. If ICs were significantly different between groups in two or more modalities, they were defined as modality-shared discriminative ICs. Notably, the terminology “discriminative” denotes statistically significant differences between groups but does not refer to differential diagnosis. Otherwise, those significantly different in only one modality were called modality-specific discriminative ICs. For ICs with significant differences, the MNI coordinates of voxels (Z > |3.5| and cluster size ≥70) were extracted by using xjView (http://www.alivelearn.net/xjview) and labeled on the AAL template.

During the analysis between the FES patients and HCs, feature matrices were normalized so that all features had the same mean sum-of-squares. The relative scaling (a normalization factor) was preserved within each modality, i.e., 0.27, 0.52, and 0.64 for GMV, fALFF, and ReHo, respectively. The number of ICs was estimated for each feature and set to 8, according to the minimum description length criteria (30) and considering calculation feasibility. Component stability was measured by repeating the infomax algorithm 10 times in ICASSO (21, 31). During the analysis between the RG and NRG, the above parameters were as follows: GMV = 0.27, fALFF = 0.52, and ReHo = 0.64 for relative scaling; IC number = 7; and repeated time of infomax algorithm = 2 in ICASSO.

In *post hoc* analyses, we investigated the correlations of mixing coefficients of the identified significant ICs with the percentage PANSS reduction by SPSS (version 24.0, IBM Corp, Armonk, USA). The Curve Estimation procedure was applied to curve estimation regression statistics. In addition, *t*-test was used to compare treatment response (the PANSS reduction) between patients with a single drug and those with multiple drugs. Pearson correlations were performed to evaluate the impacts of both illness duration on symptom severity (the PANSS score) and treatment response and CPZ equivalents on treatment response.

## Results

### Demographic and Clinical Characteristics

The demographic and clinical characteristics of the participants were shown in Table 2. The RG had more education years than the NRG (*p* = 0.01). No intergroup differences were found in age, sex, or education years between the FES patients and HCs or in age, sex, illness duration, baseline PANSS scores, or daily dosage of antipsychotics between the RG and NRG. No differences were found in the PANSS reduction between patients with a single drug and those with multiple drugs (Cohen's *d* = 0.23, *p* = 0.57). No correlations were observed between illness duration and the baseline PANSS scores (*p* = 0.71) or PANSS reduction (*p* = 0.11). CPZ equivalents did not correlate with the PANSS reduction (*p* = 0.33).

**Table 2:** Demographic and clinical characteristics of participant groups.

	**FES (*n* = 55)**	**HCs (*n* = 55)**	***P*-value**	**RG (*n* = 40)**	**NRG (*n* = 15)**	***P*-value**
Age (years)	24.7 ± 8.6	24.8 ± 8.6	0.06	23.8 ± 7.1	27.1 ± 11.6	0.32
Male/female	25/30	25/30	1.00	17/23	8/7	0.47
Education (years)	12.6 ± 2.8	12.7 ± 2.9	0.17	13.2 ± 2.6	11.1 ± 2.8	0.01
Illness duration (months)	8.6 ± 11.9	n/a		7.5 ± 9.8	11.4 ± 16.3	0.29
**Baseline PANSS**						
Positive	24.2 ± 6.4	n/a		25.1 ± 6.7	21.9 ± 5.3	0.10
Negative	18.9 ± 6.9	n/a		18.4 ± 7.1	20.4 ± 6.6	0.35
Total	91.4 ± 14.5	n/a		91.4 ± 14.3	91.6 ± 15.4	0.96
**Follow-up PANSS**						
Positive	10.7 ± 5.4	n/a		8.7 ± 3.3	16.0 ± 6.4	0.001
Negative	13.4 ± 5.5	n/a		11.4 ± 4.4	18.7 ± 4.6	<0.001
Total	51.1 ± 19.2	n/a		42.5 ± 10.0	73.9 ± 19.5	<0.001
Percentage PANSS reduction (%)	n/a	n/a		80.0 ± 14.2	29.3 ± 23.6	<0.001
CPZ equivalents (mg/day)	243.6 ± 169.2	n/a		261.6 ± 182.1	200.2 ± 128.3	0.24

*Data are expressed as the mean ± standard deviation, unless specified. FES, patients with first-episode schizophrenia; HCs, healthy controls; n/a, not applicable; RG, responder group; NRG, non-responder group; PANSS, Positive and Negative Syndrome Scale; CPZ, chlorpromazine*.

### Discriminative ICs Between FES Patients and HCs

Two-sample *t*-tests showed that mixing coefficients differed between the FES patients and HCs for three modality-specific discriminative ICs, namely, GMV-IC7, GMV-IC8, and fALFF-IC5 (Table 3). No differences were found for ReHo-ICs or across modalities (modality-shared discriminative ICs).

**Table 3:** Discriminative ICs between FES patients and HCs.

	FES (*n* = 55)^*^	HCs (*n* = 55)^*^	**Cohen's *d***	***P*-value**
GMV-IC7	0.0003 ± 0.0054	−0.0034 ± 0.0050	−0.73	<0.0010
GMV-IC8	−0.0026 ± 0.0146	0.0062 ± 0.0151	0.60	0.002
fALFF-IC5	0.0002 ± 0.0119	0.0054 ± 0.0118	0.43	0.009

* *Data are mixing coefficients, expressed as the mean ± standard deviation. FES, patients with first-episode schizophrenia; HCs, healthy controls; GMV, gray matter volume; IC, independent component; fALFF, fractional amplitude of low-frequency fluctuations*.

**Figure 1:**
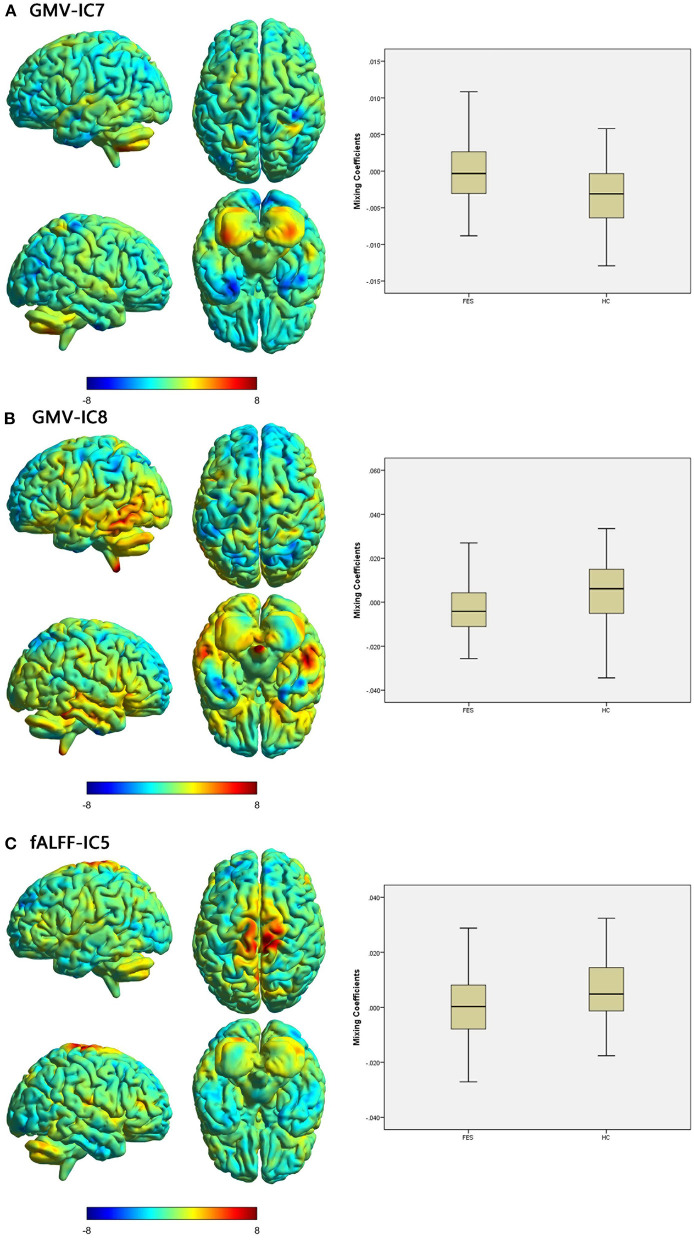
Brain maps of discriminative IC and box plots of mixing coefficients between the FES and HC groups. The left part of the figure is the brain map, and the right part is the mixing coefficient box plot (outliers have been excluded). The color bar represents the Z-scores. Box plots show that the FES group has higher mixing coefficients than the HC group for GMV-IC7 **(A)** and slightly lower mixing coefficients than the HC group for GMV-IC8 **(B)** and fALFF-IC5 **(C)**. When z values (red regions) are positive and mixing coefficients are positive, the component shows increased GMV/fALFF in the HCs. Conversely, when z values are negative (blue regions) and mixing coefficients are positive, the component shows decreased GMV/fALFF in the HCs. The opposite is true when mixing coefficients are negative. FES, patients with first-episode schizophrenia; HCs, healthy controls; IC, independent component; GMV, gray matter volume; fALFF, fractional amplitude of low-frequency fluctuations.

Specifically, the FES patients had higher mixing coefficients for GMV-IC7 (Cohen's *d* = −0.73, *p* < 0.001) but slightly lower mixing coefficients for GMV-IC8 (Cohen's *d* = 0.60, *p* = 0.002) than HCs. Brain regions with differences included the bilateral inferior temporal gyri, middle temporal gyri, calcarine, middle frontal gyri, right postcentral gyrus, and left parietal lobe (Figure 1; Supplementary Table S1). The FES patients also showed a slightly decreased mixing coefficient for fALFF-IC5 compared to the HCs (Cohen's *d* = 0.43, *p* = 0.009), involving the bilateral paracentral lobules and supplementary motor areas and the right precentral and postcentral gyri (Figure 1; Supplementary Table S1).

### Discriminative ICs Between the RG and NRG

**Figure 2:**
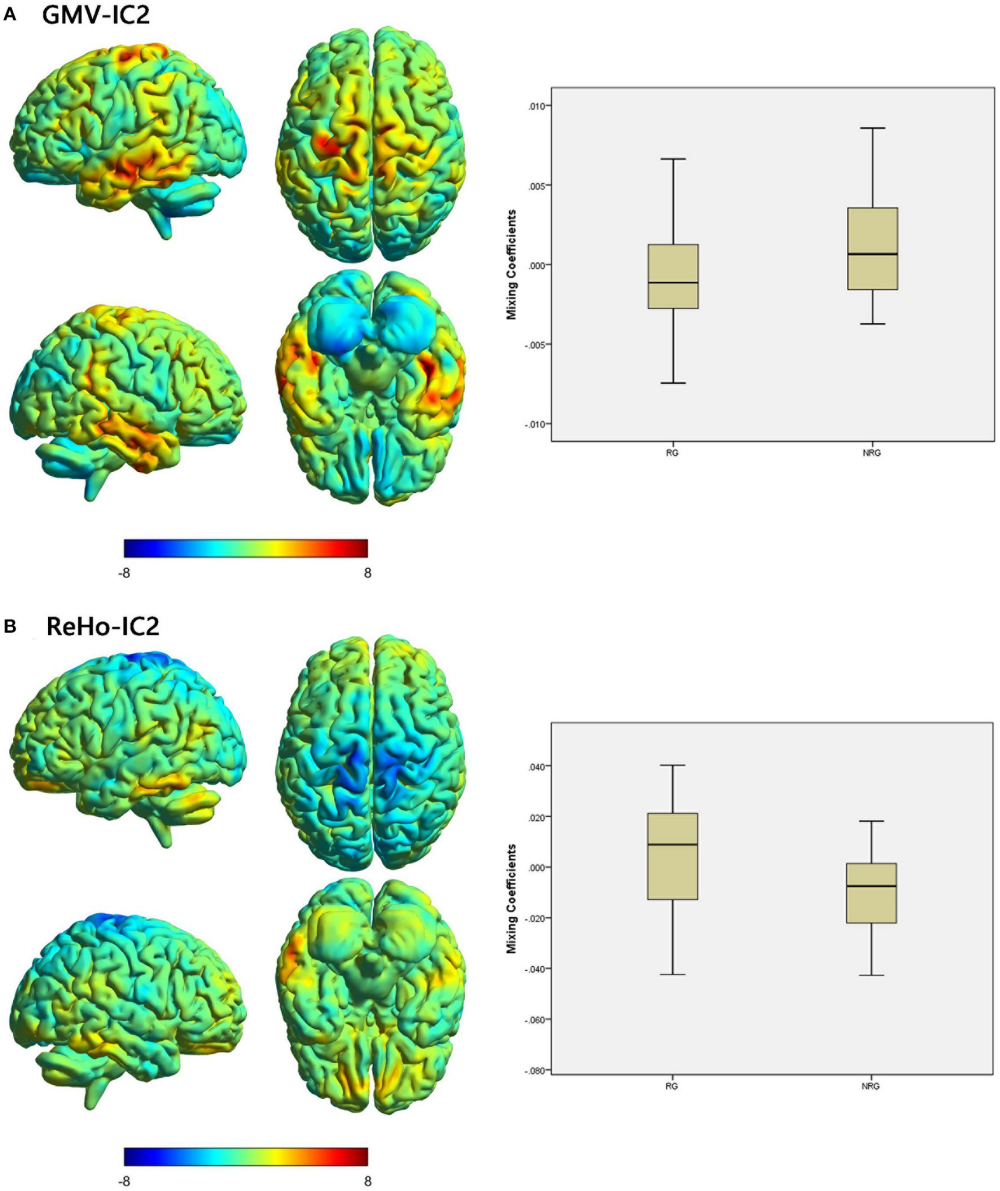
Brain maps of shared discriminative IC and box plots of mixing coefficients between the RG and NRG. The left part of the figure is the brain map, and the right part is the mixing coefficient box plot (outliers have been excluded). The color bar represents the Z-scores. Box plots show that the RG has lower mixing coefficients than the NRG for GMV-IC2 **(A)** and higher mixing coefficients than the NRG for ReHo-IC2 **(B)**. When z values (red regions) are positive and mixing coefficients are positive, the component shows increased GMV/ReHo in the NRG. Conversely, when z values are negative (blue regions) and mixing coefficients are positive, the component shows decreased GMV/ReHo in the NRG. The opposite is true when mixing coefficients are negative. RG, responder group; NRG, non-responder group; IC, independent component; GMV, gray matter volume; ReHo, regional homogeneity.

We identified one modality-shared discriminative IC between the RG and NRG (Table 4; Figure 2). The mixing coefficient was lower for GMV-IC2 (Cohen's *d* = −0.57, *p* = 0.03) but higher for ReHo-IC2 (Cohen's *d* = 0.77, *p* = 0.02) in the RG than in the NRG. The involved brain regions were predominantly in the right postcentral gyrus for ReHo-IC2 and in the bilateral temporal gyri and in the right cerebellum for GMV-IC2 (Supplementary Table S2).

**Table 4:** Discriminative ICs between the RG and NRG.

	RG (*n* = 40)^*^	NRG (*n* = 15)^*^	**Cohen's *d***	***P*-value**
**Modality-shared IC2**				
GMV	−0.0006 ± 0.0032	0.0013 ± 0.0039	−0.57	0.03
ReHo	0.0047 ± 0.0210	−0.0115 ± 0.0217	0.77	0.03
**Modality-specific**				
GMV-IC1	−0.0006 ± 0.0024	0.0015 ± 0.0026	−0.89	0.003
GMV-IC3	−0.001 ± 0.0055	0.0028 ± 0.0064	−0.67	0.001
GMV-IC6	−0.0138 ± 0.0588	0.0379 ± 0.0684	−0.84	0.02

* *Data are mixing coefficients, expressed as the mean ± standard deviation. RG, responder group; NRG, non-responder group; IC, independent component; GMV, gray matter volume; ReHo, regional homogeneity*.

**Figure 3:**
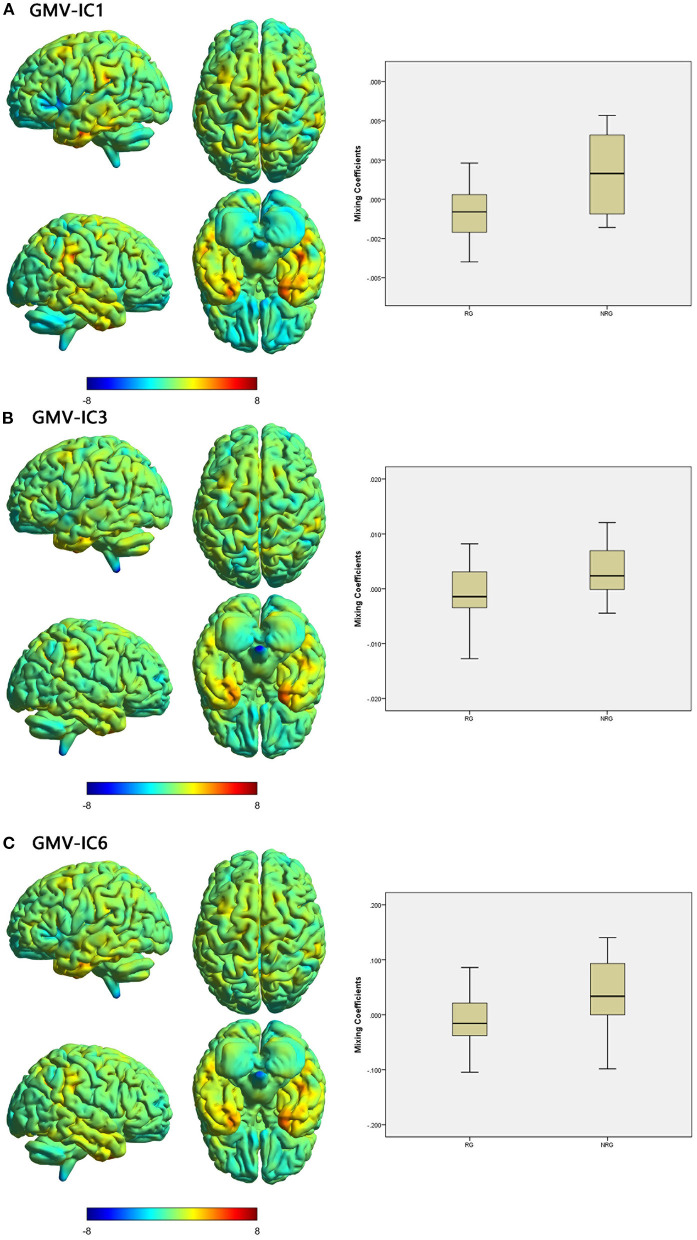
Brain maps of specific discriminative IC and box plots of mixing coefficients between the RG and NRG. The left part of the figure is the brain map, and the right part is the mixing coefficient box plot (outliers have been excluded). The color bar represents the Z-scores. Box plots show that the RG has lower mixing coefficients than the NRG for GMV-IC1 **(A)**, GMV-IC3 **(B)** and GMV-IC6 **(C)**. When z values (red regions) are positive and mixing coefficients are positive, the component shows increased GMV in the NRG. Conversely, when z values are negative (blue regions) and mixing coefficients are positive, the component shows decreased GMV in the NRG. The opposite is true when mixing coefficients are negative. RG, responder group; NRG, non-responder group; IC, independent component; GMV, gray matter volume.

Three modality-specific discriminative ICs were found (Table 4; Figure 3) that showed lower mixing coefficients for GMV-IC1 (Cohen's *d* = −0.89, *p* = 0.003), GMV-IC3 (Cohen's *d* = −0.67, *p* = 0.001), and GMV-IC6 (Cohen's *d* = −0.84, *p* = 0.02) in the RG relative to the NRG. The related brain regions mainly included the bilateral middle and inferior temporal gyri, right fusiform gyrus, left precentral gyrus, and right angular gyrus (Supplementary Table S2).

### Relationships of Mixing Coefficients With Clinical Characteristics

**Figure 4:**
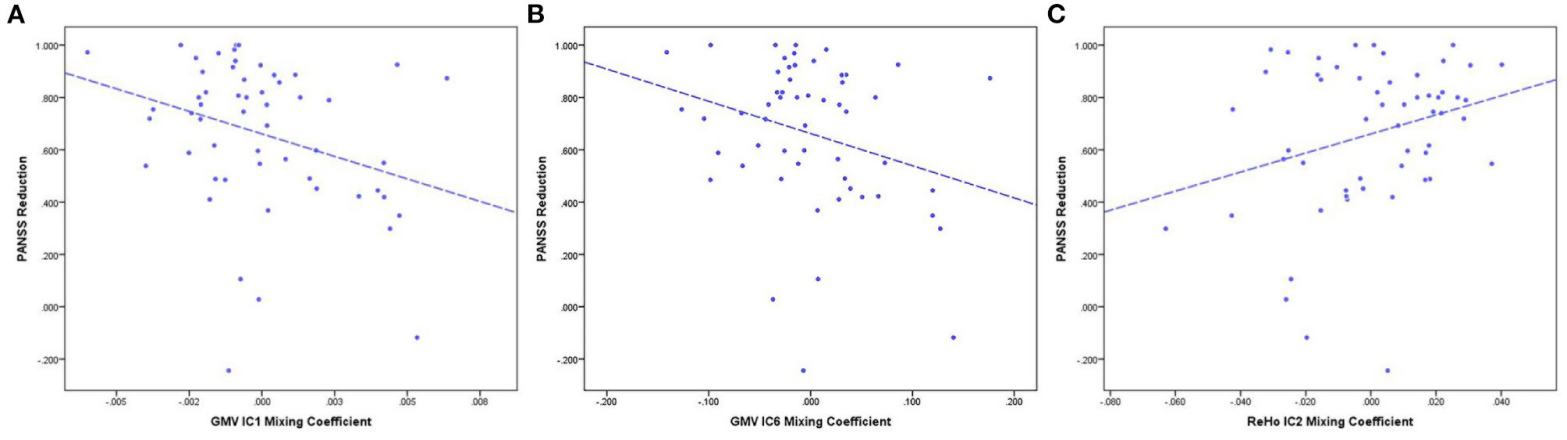
PANSS reduction and mixing coefficient correlations between the RG and NRG. **(A)** The correlation of PANSS reductions and GMV-IC1 mixing coefficients. **(B)** The correlation of PANSS reductions and GMV-IC6 mixing coefficients. **(C)** The correlation of PANSS reductions and ReHo-IC2 mixing coefficients. PANSS, Positive and Negative Syndrome Scale; RG, responder group; NRG, non-responder group; IC, independent component; GMV, gray matter volume; ReHo, regional homogeneity.

Pearson correlation analyses showed that PANSS reductions were correlated with mixing coefficients for GMV-IC1 (*r* = −0.31, *p* = 0.02), GMV-IC6 (*r* = −0.31, *p* = 0.04), and ReHo-IC2 (*r* = 0.28, *p* = 0.04) in the FES patients. A linear model was identified to provide the best curve fitting of their correlations (*p* < 0.05). As shown in Figure 4, the mixing coefficients for GMV-IC1 and GMV-IC6 were significantly negatively correlated with PANSS reductions, while the mixing coefficients for ReHo-IC2 were positively correlated with PANSS reductions.

## Discussion

With multimodal independent component analysis, we investigated the joint cortical abnormalities across anatomical and functional MRI in drug-naïve FES patients and found that different modalities identified different brain regions between non-responders and responders to 1-year antipsychotic medication based on these baseline features. Using the mCCA + jICA model, we found specific GMV and fALFF differences in drug-naïve FES patients. Notably, due to small sample size and unequal patient numbers between the responders and non-responders, our findings risk reduced statistical power. Comparing responders to 1-year antipsychotic medication with non-responders, we identified specific GMV differences, as well as shared differences between GMV and ReHo. Among the brain regions of significance, the right postcentral gyrus contributed both to modality-specific GMV features (GMV) for patient identification and modality-shared features (GMV and ReHo) for responder discrimination. The percentage reduction in PANSS scores was negatively correlated with mixing coefficients for GMV-IC1 and GMV-IC6 but positively correlated with those for ReHo-IC2. These findings indicated that joint anatomical and functional features of the cortices may reflect an early pathophysiological mechanism that is related to a 1-year treatment response.

When comparing patients with controls and responders with non-responders, there were differences identified using a structural mode and a functional mode in the right postcentral gyrus, indicating a common brain region for the diagnosis of schizophrenia and explanation of treatment effect. As part of the parietal lobe, the postcentral gyrus is called the “somatosensory cortex,” is the main receptive area of tactile and kinesthetic sensations, and plays an important role in the sensorimotor network (SMN). The SMN is involved in motor selection and execution, form perception, color processing, stereo-orientation, and depth perception (32). Deficits in the postcentral gyrus may cause persistent and aggravated disruptions within the SMN, which leads to the corresponding clinical symptoms of schizophrenia (33, 34). Our results are also consistent with the results of previous studies, which further supports the key role of the postcentral gyrus in the identification of schizophrenia (32, 35). Moreover, antipsychotic drugs such as chlorpromazine and risperidone are usually dopamine (DA) and serotonin (5-HT) receptor antagonists in the central nervous system, which have high affinity for DA and 5-HT receptors. Anatomically, the DA nigrostriatal pathway and 5-HT pathways mainly project to regions in the SMN (36). Based on anatomical relationships, DA and 5-HT participate in the regulation of large-scale network connections and play an important role in modulating SMN activity. A study showed that functional connectivity between the basal ganglia and the left pre- and postcentral gyri of healthy volunteers increased after taking levodopa compared with placebo. After taking haloperidol, functional connectivity decreased (37). Another study also showed that the blood oxygen level-dependent signal in the bilateral motor cortices of healthy young participants increased after taking levodopa (38). The present study found that the intrinsic brain activity in the right postcentral gyrus in the RG was lower than that in the NRG, which may have led to a decrease in the efficiency of the SMN and a reduction in its sensitivity to antipsychotic drugs, which manifested as clinical drug resistance. Previous studies have also shown that abnormal morphology and functional activity in this area is important in the pathogenesis of schizophrenia. Compared with an NRG, Anderson et al. reported that an RG had decreased GMV in the postcentral gyrus (3, 13). A recent study demonstrated that, compared with an NRG, an RG had decreased ALFF values (39) in the left postcentral gyrus. Together, these results suggest that the postcentral gyrus can be used as a biomarker for the diagnosis of schizophrenia and differentiating responses to treatment at the neuroanatomical and functional levels.

In addition, there were several relatively large regions (number of regional voxels >70) from the GMV features that were significant in different therapeutic effect groups, including the bilateral supplementary motor areas, the bilateral thalami, the left insula, the left superior occipital gyrus, the left postcentral gyrus, the right superior temporal gyrus, and particular regions of the cerebellum. The significant correlations between the GMV-IC1, GMV-IC6, and ReHo-IC2 mixing coefficients and the PANSS reduction percentages may represent the role of the GMV mainly in the parietal, frontal, and temporal lobes and brain activity in the postcentral gyrus in the treatment response in patients with schizophrenia. A previous systematic review of 19 studies also showed the same robust results: RG patients had more extensive GMV reductions than NRG patients (40). There was an interesting finding that the change in cerebellar GMV was also one of the regions related to therapeutic effects. Some studies have suggested that patients with schizophrenia have structural defects in the cerebellum (41–43). The cerebellum seems to play the role of a universal modulator, which can detect pattern changes and errors in movement and thinking (44) and provide adaptive feedback to the cerebral cortex (45–47). A study based on a voxel-based morphological analysis to assess GMV found that there was a potential causal relationship with an aberrant prefrontal-thalamic-cerebellar circuit (48). A neuroimaging study of the basal ganglia in patients with schizophrenia before and after the use of antipsychotic drugs for 6 months found that the functional connections between the bilateral thalami and cerebellum were reduced after treatment. Therefore, the cerebellum is also very important in the assessment of therapeutic effects. However, it is not clear whether the change is primary or secondary to cerebrum changes.

The abnormalities in modality-shared IC indicated that these significantly different brain regions across the two corresponding modalities were potentially correlated. ReHo-IC2 showed that the intrinsic activity of the right postcentral gyrus in the RG was lower than that in the NRG, and GMV-IC2 showed a decrease in extensive range of brain regions, mainly distributed in the default-mode network (DMN). Our previous study found that changes in GMV in patients with schizophrenia can disrupt brain functional connectivity (49). Our results further extend the previous findings that this interaction also exists in explaining differential responsiveness to the therapeutic effects. In general, functional MRI reflects physiological changes associated with acute psychosis, while brain anatomical changes reflect more stable and long-term changes (50). However, because this study is a cross-sectional study, the causality in this interaction is not clear. Although our results cannot determine the sequence of changes in structural and functional different brain regions, some pathophysiological and neuroimaging studies have shown that there is a close relationship between these regions. As explained above, DA and 5-HT can project to the SMN, and they can also project to the DMN; these neurotransmitters can regulate functional connections and fALFF in both the SMN and DMN (36). In addition, an imbalance in excitability between the SMN and DMN will cause positive and negative symptoms of schizophrenia (51). For example, when the DMN is repressive and the SMN is relatively active, the patient's manifestations are mainly manic and other positive symptoms. Conversely, if the SMN is repressive and the DMN is relatively active, the patient's manifestations are mainly negative symptoms such as depression. Currently available antipsychotic drugs (mostly selective monoaminergic antagonists) usually improve the clinical symptoms of patients by regulating the transmission of these neurotransmitters. Structural or functional defects in these brain regions may change the ability of therapeutic drugs to regulate neurotransmitters, thus reducing the treatment effect. At the neuroimaging level, a recent study found that internetwork integration between the DMN and SMN was a key negative predictor for the improvements in clinical symptoms after antipsychotic treatment (52). Our study further suggested that the increased correlation between brain activity and gray matter deficits between the two networks is of great significance in different clinical outcomes.

Our study has some limitations. First, the drug selection and medication dosage were not controlled in the study. It is mainly attributed to the heterogeneity in antipsychotic drugs and small sample size of our study, which impeded further stratification analyses. While controlling for these factors is extremely difficult during 1-year follow-ups, this may nevertheless have complicated the interpretation of our results. Second, due to the small sample size of this study, to include as many subjects as possible, there was a significant difference in the years of education, and the number of subjects was not balanced between the RG and NRG. The small sample size and unequal number between the responders and non-responders would reduce the statistical power of our study. Therefore, the results of this study merit replication in future studies with larger sample size and stratification analysis.

## Conclusions

In summary, by combining structural and functional MRI in drug-naïve FES patients, our study revealed that structural and functional deficits in multiple regions of the brain at baseline were related to a poorer response to antipsychotic treatment at the 1-year follow-up and thus may serve as a potential biomarker for long-term treatment outcomes. In addition, there were interactions between some significantly different brain regions in anatomical and functional modalities. Our findings deepen the understanding of brain mechanisms underlying heterogeneity in treatment outcomes in schizophrenia and provide evidence for the utility of combining structural and functional imaging in differentiating 1-year treatment responses in drug-naïve FES patients.

## Data Availability Statement

The raw data supporting the conclusions of this article will be made available by the authors, without undue reservation.

## Ethics Statement

The studies involving human participants were reviewed and approved by West China Hospitals research Ethics Committee. The patients/participants provided their written informed consent to participate in this study.

## Author Contributions

Conception and design by CY, NH, HC, QG, and SL. Administrative support by CY, NH, WZ, QG, and SL. Provision of study materials or patients by CY, NH, BT, WZ, YX, and SL. Collection and assembly of data by CY, NH, BT, WZ, YZ, and YX. Data analysis and interpretation by CY, NH, HC, BT, WZ, YZ, and YX. All authors contributed to the article and approved the submitted version.

## Funding

This study was supported by the National Natural Science Foundation of China (Project Nos. 82102000, 82071908, 81820108018, and 81621003), Sichuan Science and Technology Program (Project Nos. 2021JDTD0002, 2020YJ0018, and 2019YJ0155), Science and Technology Project of the Health Commission of Sichuan Province (Project No. 20PJ010), Fundamental Research Funds for the Central Universities (Project No. 2020SCU12053), Postdoctoral Interdisciplinary Research Project of Sichuan University (Project No. 0040204153248), 1.3.5 Project for Disciplines of Excellence, West China Hospital, Sichuan University (Project Nos. ZYYC08001 and ZYJC18020), Post-Doctor Research Project, West China Hospital, Sichuan University (Project No. 2020HXBH005), and the University of Cincinnati Schizophrenia Research Fund. SL acknowledges the support from the Humboldt Foundation Friedrich Wilhelm Bessel Research Award.

## Conflict of Interest

The authors declare that the research was conducted in the absence of any commercial or financial relationships that could be construed as a potential conflict of interest.

## Publisher's Note

All claims expressed in this article are solely those of the authors and do not necessarily represent those of their affiliated organizations, or those of the publisher, the editors and the reviewers. Any product that may be evaluated in this article, or claim that may be made by its manufacturer, is not guaranteed or endorsed by the publisher.
