# Supplementary material for: A Multimodal Fusion Analysis of Pretreatment Anatomical and Functional Cortical Abnormalities in Responsive and Non-responsive Schizophrenia
Source: Front Psychiatry. 2021 Dec 1;12:737179. doi: 10.3389/fpsyt.2021.737179 (PMC8671303; doi:10.3389/fpsyt.2021.737179)
Supplement: Supplementary file 1 [file Data_Sheet_1.docx]

**Table S1. Brain regions of discriminative ICs between FES patients and HCs.**

| **IC** | **Cluster** | **Regions of cluster** | **Number of voxels** | **Peak region** | **Max Z value (x, y, z)** |
| --- | --- | --- | --- | --- | --- |
| GMV-IC7 |  |  |  |  |  |
| Positive |  |  |  |  |  |
|  | 1 | Right cerebellum 8 | 428 | Right cerebellum 8 | 4.78 (34.5, -57, -51) |
|  | 2 | Left cerebellum 8 | 659 | Left cerebellum 8 | 5.47 (-34.5, -51, -51) |
|  | 3 | Left precentral gyrus | 94 | Left precentral gyrus | 4.35 (-42, 1.5, 33) |
| Negative |  |  |  |  |  |
|  | 4 | Right inferior temporal gyrus, right fusiform, and right para-hippocampal gyrus | 805 | Right inferior temporal gyrus | -5.70 (43.5, -7.5, -40.5) |
|  | 5 | Left inferior temporal gyrus, left fusiform, and left middle temporal pole | 803 | Left inferior temporal gyrus | -5.88 (-25.5, 1.5, -42) |
|  | 6 | Left hippocampus | 82 | Left hippocampus | -3.94 (-19.5, -13.5, -19.5) |
|  | 7 | Left middle temporal gyrus | 85 | Left middle temporal gyrus | -4.49 (-51, -7.5, -13.5) |
|  | 8 | Right calcarine, left calcarine, right lingual gyrus, left cuneus | 2823 | Right calcarine | -5.44 (10.5, -79.5, 7.5) |
|  | 9 | Right middle temporal gyrus, right middle occipital gyrus, right angular gyrus, right inferior occipital gyrus | 2517 | Right middle temporal gyrus | -6.00 (45, -70.5, 18) |
|  | 10 | Triangular part of left inferior frontal gyrus, and orbital part of left middle frontal gyrus | 811 | Triangular part of left inferior frontal gyrus | -5.38 (-37.5, 37.5, 12) |
|  | 11 | Left calcarine | 297 | Left calcarine | -4.70 (-19.5, -61.5, 6) |
|  | 12 | Right middle frontal gyrus | 175 | Right middle frontal gyrus | -5.52 (37.5, 43.5, 7.5) |
|  | 13 | Left supramarginal gyrus | 271 | Left superior temporal gyrus | -4.84 (-51, -34.5, 19.5) |
|  | 14 | Triangular part of right inferior frontal gyrus | 189 | Triangular part of right inferior frontal gyrus | -5.09 (43.5, 27, 21) |
|  | 15 | Left middle frontal gyrus | 454 | Left middle frontal gyrus | -6.95 (-34.5, 22.5, 37.5) |
|  | 16 | Left middle temporal gyrus, left angular gyrus, left middle occipital gyrus | 1525 | Left middle temporal gyrus | -5.92 (-40.5, -54, 19.5) |
|  | 17 | Right precuneus, right middle cingulum | 298 | Right precuneus | -4.19 (4.5, -55.5, 31.5) |
|  | 18 | Left supramarginal gyrus | 72 | Left supramarginal gyrus | -4.18 (-58.5, -24, 39) |
|  | 19 | Left precuneus, left middle cingulum | 711 | Left precuneus | -5.12 (-6, -45, 49.5) |
|  | 20 | Right postcentral gyrus, right supramarginal gyrus | 346 | Right postcentral gyrus | -6.15 (36, -33, 43.5) |
|  | 21 | Right precentral gyrus | 111 | Right precentral gyrus | -4.56 (36, 0, 48) |
|  | 22 | Right postcentral gyrus | 297 | Right postcentral gyrus | -6.29 (40.5, -31.5, 63) |
|  | 23 | Right postcentral gyrus | 73 | Right postcentral gyrus | -4.53 (16.5, -43.5, 72) |
| GMV-IC8 |  |  |  |  |  |
| Positive |  |  |  |  |  |
|  | 1 | Left cerebellum 8 | 275 | Left cerebellum 8 | 4.89 (-34.5, -60, -55.5) |
|  | 2 | Left cerebellum crus1 | 115 | Left cerebellum crus1 | 4.42 (-40.5, -75, -33) |
|  | 3 | Right inferior temporal gyrus | 1322 | Right inferior temporal gyrus | 7.04 (51, -34.5, -24) |
|  | 4 | Left middle and inferior temporal gyrus, left middle and inferior occipital gyrus | 2483 | Left middle temporal gyrus | 10.16 (-45, -63, 3) |
|  | 5 | Orbital part of right inferior frontal gyrus | 81 | Orbital part of right inferior frontal gyrus | 4.12 (21, 15, -22.5) |
|  | 6 | Left cerebellum 4 and 5 | 71 | Left cerebellum 4 and 5 | 3.88 (-13.5, -42, -19.5) |
|  | 7 | Left inferior temporal gyrus | 77 | Left inferior temporal gyrus | 4.52 (-42, -42, -18) |
|  | 8 | Right inferior temporal gyrus | 178 | Right inferior temporal gyrus | 6.09 (45, -55.5, -10.5) |
|  | 9 | Left middle temporal gyrus | 91 | Left middle temporal gyrus | 3.97 (-60, -10.5, -12) |
|  | 10 | Right middle temporal gyrus | 203 | Right middle temporal gyrus | 4.85 (66, -24, -7.5) |
|  | 11 | Left middle and inferior occipital gyrus | 641 | Left middle occipital gyrus | 7.05 (-25.5, -90, 1.5) |
|  | 12 | Orbital part of right inferior frontal gyrus | 92 | Orbital part of right inferior frontal gyrus | 4.60 (49.5, 25.5, -3) |
|  | 13 | Right calcarine | 79 | Right calcarine | 4.42 (10.5, -88.5, 0) |
|  | 14 | Right calcarine | 133 | Right calcarine | 4.78 (25.5, -55.5, 6) |
|  | 15 | Triangular part of right inferior frontal gyrus, opercular part of right inferior frontal gyrus | 476 | Triangular part of right inferior frontal gyrus | 6.74 (51, 13.5, 21) |
|  | 16 | Left calcarine | 75 | Left calcarine | 4.26 (-16.5, -64.5, 15) |
|  | 17 | Right precentral and postcentral gyrus, right supramarginal gyrus | 651 | Right precentral gyrus | 5.12 (55.5, -12, 43.5) |
|  | 18 | Right inferior parietal gyrus | 204 | Right inferior parietal gyrus | 8.40 (31.5, -46.5, 40.5) |
| Negative |  |  |  |  |  |
|  | 19 | Right fusiform | 150 | Right fusiform | -5.54 (24, 6, -46.5) |
|  | 20 | Right cerebellum 8 | 155 | Right cerebellum 8 | -4.66 (33, -51, -45) |
|  | 21 | Left inferior temporal gyrus | 145 | Left inferior temporal gyrus | -5.09 (-43.5, -9, -40.5) |
|  | 22 | Right inferior temporal gyrus | 92 | Right inferior temporal gyrus | -4.76 (45, -7.5, -43.5) |
|  | 23 | Left superior and middle frontal gyrus, orbital part of left superior and middle frontal gyrus | 762 | Left superior frontal gyrus | -5.46 (-24, 54, 3) |
|  | 24 | Right middle temporal gyrus | 271 | Right middle temporal gyrus | -8.19 (48, -52.5, 7.5) |
|  | 25 | Triangular part of left inferior frontal gyrus | 108 | Triangular part of left inferior frontal gyrus | -4.85 (-37.5, 39, 10.5) |
|  | 26 | Medial part of right superior frontal gyrus | 75 | Medial part of right superior frontal gyrus | -4.02 (18, 64.5, 16.5) |
|  | 27 | Right angular gyrus, right middle and superior occipital gyrus, right superior parietal gyrus | 1025 | Right angular gyrus | -6.48 (34.5, -57, 42) |
|  | 28 | Right middle frontal gyrus | 147 | Right middle frontal gyrus | -6.72 (39, 31.5, 19.5) |
|  | 29 | Right middle frontal gyrus | 138 | Right middle frontal gyrus | -4.18 (33, 46.5, 27) |
|  | 30 | Left middle occipital gyrus, left superior parietal gyrus | 574 | Left middle occipital gyrus | -6.73 (-25.5, -66, 34.5) |
|  | 31 | Left middle frontal gyrus | 75 | Left middle frontal gyrus | -5.78 (-33, 31.5, 30) |
|  | 32 | Left supramarginal gyrus, left inferior parietal gyrus | 946 | Left supramarginal gyrus | -5.92 (-55.5, -33, 33) |
|  | 33 | Left middle and superior frontal gyrus | 355 | Left frontal middle gyrus | -6.49 (-21, 40.5, 31.5) |
|  | 34 | Left precuneus | 309 | Left precuneus | -4.83 (-1.5, -61.5, 42) |
|  | 35 | Right middle frontal gyrus | 155 | Right middle frontal gyrus | -4.75 (33, 30, 48) |
|  | 36 | Left precentral gyrus | 309 | Left precentral gyrus | -5.66 (-40.5, -4.5, 48) |
|  | 37 | Right precuneus | 80 | Right precuneus | -4.56 (10.5, -76.5, 51) |
|  | 38 | Right precuneus | 108 | Right precuneus | -5.00 (12, -42, 52.5) |
|  | 39 | Right superior and middle frontal gyrus, right precentral gyrus | 875 | Right superior frontal gyrus | -6.62 (18, -12, 64.5) |
| fALFF-IC5 |  |  |  |  |  |
| Positive |  |  |  |  |  |
|  | 1 | Left para-central lobule | 93 | Left para-central lobule | 10.19 (-13, -28, 75) |

Each independent component is converted to Z-scores. In each cluster, the maximum Z value and its Montreal Neurological Institute coordinates are listed. Abbreviations: FES, patients with first episode schizophrenia; HCs, healthy controls; IC, independent component; GMV, gray matter volume; fALFF, fractional amplitude of low-frequency fluctuations.

**Table S2. Brain regions of discriminative ICs between RG and NRG.**

| **IC** | **Cluster** | **Regions of cluster** | **Number of voxels** | **Peak region** | **Max Z value (x, y, z)** |
| --- | --- | --- | --- | --- | --- |
| Modality-shared IC2 |  |  |  |  |  |
| GMV |  |  |  |  |  |
| Positive |  |  |  |  |  |
|  | 1 | Right middle and inferior temporal gyrus | 2149 | Right middle temporal gyrus | 6.82 (63, -9, -19.5) |
|  | 2 | Right inferior temporal gyrus, right fusiform | 587 | Right inferior temporal gyrus | 8.00 (43.5, -31.5, -22.5) |
|  | 3 | Left inferior and middle temporal gyrus, left fusiform | 4422 | Left inferior temporal gyrus | 7.60 (-43.5, -33, -22.5) |
|  | 4 | Right middle occipital gyrus | 111 | Right middle occipital gyrus | 4.46 (39, -75, 7.5) |
|  | 5 | Left calcarine | 135 | Left calcarine | 5.04 (-22.5, -58.5, 4.5) |
|  | 6 | Right calcarine | 237 | Right calcarine | 5.86 (-27, -54, 6) |
|  | 7 | Left angular gyrus, left middle temporal gyrus | 456 | Left angular gyrus | 6.01 (-43.5, -52.5, 22.5) |
|  | 8 | Left middle and superior occipital gyrus, left superior parietal gyrus | 915 | Left middle occipital gyrus | 8.88 (-25.5, -64.5, 33) |
|  | 9 | Right supramarginal gyrus, right superior temporal gyrus | 1093 | Right supramarginal gyrus | 8.08 (55.5, -40.5, 30) |
|  | 10 | Left middle frontal gyrus, triangular part of left inferior frontal gyrus, left precentral gyrus | 1667 | Left middle frontal gyrus | 8.40 (-36, 15, 42) |
|  | 11 | Right middle occipital gyrus | 249 | Right middle occipital gyrus | 5.77 (31.5, -66, 28.5) |
|  | 12 | Left supramarginal gyrus | 212 | Left supramarginal gyrus | 5.42 (-55.5, -33, 25.5) |
|  | 13 | Opercular part of right inferior frontal gyrus, right middle frontal gyrus, right precentral gyrus | 1084 | Opercular part of right inferior frontal gyrus | 7.32 (39, 6, 33) |
|  | 14 | Left middle frontal gyrus | 127 | Left middle frontal gyrus | 5.10 (-24, 10.5, 48) |
|  | 15 | Left precentral and postcentral gyrus, left para-central lobule, left supplementary motor area, left precuneus, left superior frontal gyrus | 1682 | Left precentral gyrus | 6.58 (-30, -18, 67.5) |
|  | 16 | Right supplementary motor area, right superior frontal gyrus | 530 | Right supplementary motor area | 6.00 (13.5, -3, 67.5) |
|  | 17 | Right postcentral gyrus | 125 | Right postcentral gyrus | 5.36 (12, -36, 75) |
| Negative |  |  |  |  |  |
|  | 18 | Right cerebellum 8 and 9, left cerebellum 8 and 9 | 2520 | Right cerebellum 9 | -8.00 (7.5, -55.5, -57) |
|  | 19 | Right para-hippocampal gyrus | 72 | Right para-hippocampal gyrus | -4.63 (33, -39, -10) |
|  | 20 | Vermis 4 and 5, right cerebellum 4 and 5 | 180 | Vermis 4 and 5 | -4.23 (6, -48, -12) |
|  | 21 | Right thalamus | 316 | Right thalamus | -5.45 (9, -15, 10.5) |
|  | 22 | Left superior occipital gyrus | 72 | Left superior occipital gyrus | -4.73 (-9, -97.5, 12) |
|  | 23 | Left thalamus | 94 | Left thalamus | -4.44 (-9, -16.5, 12) |
|  | 24 | Right middle temporal gyrus | 99 | Right middle temporal gyrus | -5.16 (40.5, -63, 16.5) |
|  | 25 | Right superior occipital gyrus | 98 | Right superior occipital gyrus | -4.14 (24, -87, 19) |
|  | 26 | Left inferior parietal gyrus | 138 | Left inferior parietal gyrus | -5.30 (-28.5, -51, 42) |
| ReHo |  |  |  |  |  |
| Negative |  |  |  |  |  |
|  | 1 | Right postcentral gyrus | 76 | Right postcentral gyrus | -5.37 (18, -33, 75) |
| Modality-specific |  |  |  |  |  |
| GMV-IC1 |  |  |  |  |  |
| Positive |  |  |  |  |  |
|  | 1 | Right fusiform, temporal pole of right middle temporal gyrus, right inferior temporal gyrus | 573 | Right fusiform | 4.76 (27, 4.5, -43.5) |
|  | 2 | Left fusiform | 70 | Left fusiform | 4.59 (-28.5, 6, -42) |
|  | 3 | Left inferior temporal gyrus | 202 | Left inferior temporal gyrus | 5.45 (-51, -7.5, -28.5) |
|  | 4 | Right fusiform, right inferior temporal gyrus | 233 | Right fusiform | 5.00 (46.5, -31.5, -24) |
|  | 5 | Right inferior temporal gyrus | 277 | Right inferior temporal gyrus | 5.12 (52.5, -48, -19) |
|  | 6 | Left inferior temporal gyrus | 73 | Left inferior temporal gyrus | 4.03 (-57, -33, -19.5) |
|  | 7 | Left middle temporal gyrus, left angular gyrus | 447 | Left middle temporal gyrus | 5.56 (-45, -51, 16.5) |
|  | 8 | Right supramarginal gyrus | 751 | Right supramarginal gyrus | 7.33 (57, -40.5, 27) |
|  | 9 | Left supramarginal gyrus | 288 | Left supramarginal gyrus | 5.64 (-57, -30, 28.5) |
|  | 10 | Right angular gyrus | 572 | Right angular gyrus | 7.39 (42, -57, 33) |
|  | 11 | Right precentral gyrus | 84 | Right precentral gyrus | 4.07 (48, 1.5, 46.5) |
|  | 12 | Left middle frontal gyrus | 70 | Left middle frontal gyrus | 4.61 (-34.5, 13.5, 48) |
| Negative |  |  |  |  |  |
|  | 13 | Right cerebellum 9 | 189 | Right cerebellum 9 | -4.77 (7.5, -54, -58.5) |
|  | 14 | Left cerebellum 4 and 5, vermis 4 and 5 | 309 | Left cerebellum 4 and 5 | -4.31 (-6, -48, -12) |
|  | 15 | Right fusiform | 118 | Right fusiform | -5.10 (24, -78, -9) |
|  | 16 | Left insula | 348 | Left insula | -4.62 (-33, 18, 4.5) |
| GMV_IC3 |  |  |  |  |  |
| Positive |  |  |  |  |  |
|  | 1 | Right fusiform, temporal pole of right middle temporal gyrus | 363 | Right fusiform | 4.69 (25.5, 6, -42) |
|  | 2 | Left temporal middle gyrus | 76 | Left middle temporal gyrus | -5.08 (-39, -58.5, 19.5) |
|  | 3 | Right angular gyrus | 233 | Right angular gyrus | 6.13 (42, -57, 31.5) |
| Negative |  |  |  |  |  |
|  | 4 | Right cerebellum 9 | 147 | Right cerebellum 9 | -7.35 (0, -46.5, -69) |
|  | 5 | Right middle temporal gyrus | 188 | Right middle temporal gyrus | -5.08 (45, -64.5, 1.5) |
|  | 6 | Opercular part of right inferior frontal gyrus | 133 | Opercular part of right inferior frontal gyrus | -5.32 (39, 13.5, 31.5) |
| GMV_IC6 |  |  |  |  |  |
| Positive |  |  |  |  |  |
|  | 1 | Right fusiform, temporal pole of right middle temporal gyrus | 344 | Right fusiform | 4.52 (27, 4.5, -43.5) |
|  | 2 | Left middle temporal gyrus | 135 | Left middle temporal gyrus | 4.13 (-42, -55, 18) |
|  | 3 | Right supramarginal gyrus | 96 | Right supramarginal gyrus | 4.87 (58.5, -40.5, 24) |
|  | 4 | Right angular gyrus | 288 | Right angular gyrus | 6.11 (42, -57, 33) |
| Negative |  |  |  |  |  |
|  | 5 | Right cerebellum 9 | 110 | Right cerebellum 9 | -4.87 (1.5, -46.5, -67.5) |

Each independent component is converted to Z-scores. In each cluster, the maximum Z value and its Montreal Neurological Institute coordinates are listed. Abbreviation: RG, responder group; NRG, nonresponder group; IC, independent component; GMV, gray matter volume; ReHo, regional homogeneity.
